# Stability of Synchronization Clusters and Seizurability in Temporal Lobe Epilepsy

**DOI:** 10.1371/journal.pone.0041799

**Published:** 2012-07-23

**Authors:** Agostina Palmigiano, Jesús Pastor, Rafael García de Sola, Guillermo J. Ortega

**Affiliations:** 1 Instituto de Investigación Sanitaria Hospital de la Princesa, Madrid, Spain; 2 Physics Department, Universidad de Buenos Aires, Buenos Aires, Argentina; National Research & Technology Council, Argentina

## Abstract

**Purpose:**

Identification of critical areas in presurgical evaluations of patients with temporal lobe epilepsy is the most important step prior to resection. According to the “epileptic focus model”, localization of seizure onset zones is the main task to be accomplished. Nevertheless, a significant minority of epileptic patients continue to experience seizures after surgery (even when the focus is correctly located), an observation that is difficult to explain under this approach. However, if attention is shifted from a specific cortical location toward the network properties themselves, then the epileptic network model does allow us to explain unsuccessful surgical outcomes.

**Methods:**

The intraoperative electrocorticography records of 20 patients with temporal lobe epilepsy were analyzed in search of interictal synchronization clusters. Synchronization was analyzed, and the stability of highly synchronized areas was quantified. Surrogate data were constructed and used to statistically validate the results. Our results show the existence of highly localized and stable synchronization areas in both the lateral and the mesial areas of the temporal lobe ipsilateral to the clinical seizures. Synchronization areas seem to play a central role in the capacity of the epileptic network to generate clinical seizures. Resection of stable synchronization areas is associated with elimination of seizures; nonresection of synchronization clusters is associated with the persistence of seizures after surgery.

**Discussion:**

We suggest that synchronization clusters and their stability play a central role in the epileptic network, favoring seizure onset and propagation. We further speculate that the stability distribution of these synchronization areas would differentiate normal from pathologic cases.

## Introduction

Partial epileptic seizures were traditionally thought to originate in specific areas of the cortex known as seizure onset zones (SOZ), before spreading to other areas, known as epileptogenic zones (EZ), some of which overlap with the SOZ. EZ are essential for seizures to propagate [1], [2]. Surgical approaches to this condition include resection or disconnection of these areas, principally the SOZ (usually identified as the epileptic focus), from the rest of the brain. This “single focus” model has been challenged [3]–[5] in favor of a network model in which the focus (or foci) would be distributed along the limbic structures.

S.S. Spencer [6] proposed a different network view, in which emphasis shifts from the epileptic focus (or foci) toward the properties of the network itself, suggesting that studies should focus on the epileptic network as a whole and not on the specific SOZ (see also [7]). Surgery for temporal lobe epilepsy (TLE) targets a wide area, in which multiple rather than single structures are resected (mainly tailored corticectomy and extended amygdalohippocampectomy) [8], [9]. It is difficult to determine whether the absence of postoperative seizures is a consequence of the resection (or disconnection) of the focus–which presumably has been correctly localized–or of the destruction of the network topology. Moreover, even if the suspected epileptogenic area is correctly localized in presurgical studies and resected during surgery, a significant minority of patients continue to experience seizures after surgery, thus favoring the concept of an epileptic network.

In recent years, major advances have been made in the study of complex networks [10], [11], [12] and in the dynamics of underlying network topologies [13]. Results for the onset and stability of synchronization in oscillatory networks [14] are of paramount importance for the study of epilepsy. Complex network approaches are now actively applied in the analysis of epileptic data recorded using various neurophysiological techniques [15], [16], [17] and functional magnetic resonance imaging (fMRI) studies [18], which attempt to uncover the structural characteristics of the epileptic network. However, little attention has been paid to the temporal dimension of the network, in terms of either formal studies or their applications. Recent advances in time-varying graphs [19] show that small world behavior [20], a network characteristic which favors faster and stable synchronization may be present, even in cases where the underlying topology lacks this property.

Reports of locally hyper-synchronous cortical areas have been published recently by several independent groups [7], [21], [22]. In the present study, a step forward is accomplished by showing that local synchronization (LS) areas also display the smallest temporal variability. Furthermore, we will show that resection of these stable LS areas are associated with a favorable surgery outcome. Results presented here clearly demonstrate that, contrary to what could be expected, LS areas do not correlate with SOZ.

Although the EZ is commonly considered indispensable for the generation of clinical seizures [2], it was traditionally associated with the SOZ [23], [24]. Consequently, the word epileptogenicity was associated with the capacity of particular areas, or even whole structures, to initiate seizures [24]. In order to avoid misunderstandings, we use the term ictogenicity of the epileptic network as the capacity of the network to seize, without specific reference to the SOZ. Moreover, because it is usual to define synchronizability of a complex network as the capacity to generate and sustain global synchronization [14]; the term “seizurability” should be equivalently used as ictogenicity.

## Methods

### Patients

The study sample comprised 20 drug-resistant TLE patients (11 females) who underwent surgery at the Epilepsy Unit of Hospital de La Princesa (Table 1). Patients gave their written informed consent, and the Comité ético de investigación clínica del Hospital de la Princesa (the local ethics committee) approved the study. Patients were evaluated intraoperatively with 4×5 subdural electrode grids (G1–G20, interelectrode distance, 1 cm) and a 1×8 electrode mesial strip (S1–S8, interictal distance, 1 cm), under low doses of sevoflurane (0.5%) and remifentanil (0.1 mg/kg/min). During recordings, the anesthetic level was stabilized by maintaining the bispectral index at values in the range of 55–60 [25]. The grid was placed over the lateral temporal cortex, with the border parallel to the sylvian fissure and covering gyri T1–T3 and, sometimes, T4. The grid position was recorded with a video camera or photographed. As in [21], the reference electrode was placed on the contralateral ear and in some cases was moved to the nearby scalp in order to verify that no common reference electrode contamination exists, an issue especially important when phase synchronization measures are used [26]. No reformatting of data (e.g. Laplacian operator) has been performed to deal with volume conduction due its low effect when recording from the cortical surface [27]. The orientation of the electrode grid (parallel or perpendicular to the anterior–posterior temporal lobe axis) varied from patient to patient due to surgical constraints (e.g., size of craniotomy, temporal lobe atrophy, localization of veins). Presurgical evaluation was performed according to the protocol of Hospital La Princesa, as previously reported [28]. Temporal lobe lateralization has been carried out by v-EEG, which consist of scalp EEG plus Foramen Ovale Electrodes (FOE) and video recording. Interictal paroxysmal activity and seizure onset information was also used to assess whether ictogenic areas were located at the lateral or at the mesial side of the temporal lobe, as displayed in Table 1, v-EEG column. Surgery was guided by raw electrocorticogram (ECoG) data and other presurgical information, such as video electroencephalogram and magnetic resonance imaging. Portions of the lateral temporal cortex, the amygdala, and the hippocampus were excised using anterior medial temporal resection tailored to the ECoG [29].

**Table 1:** Patients’ clinical data.

Patient	Gender	Patient age/time evolution (years)	Freq	V-EEG	MRI	Surgery/Outcome(1 year)	Success	CV loc
A	M	41/11	d	L Mes	Normal	L AMTR/IB	1	LLL
B	F	23/12	d	R Mes	Lat R T Lesion	R AMTR/IA	1	LLL
C	F	48/32	d	R Mes	R HS	R AMTR/IA	1	MLL
D	F	26/14	d	L Mes	Normal	L AMTR/IA	1	LLL
E	F	22/22	d	L Mes	L atrophy	L AMTR/IA	1	MLM
**F**	**M**	**28/17**	**d**	**L Lat**	**Normal**	**L Cort/III**	**0**	**MMM**
G	M	44/29	w	L Mes	Normal	L AMTR/IA	1	LLL
H	M	42/9	m	R Mes	L Mes alteration	L AMTR/IA	1	LLM
I	M	23/22	w	L Mes	L HS	L AMTR/IC	1	MMM
J	M	34/25	w	L Mes	L T A-M Tumor	L AMTR/IA	1	LLL
**K**	**F**	**48/32**	**w**	**R Mes**	**R HS**	**R AMTR/II**	**0**	**MLL**
L	F	32/30	w	L Mes	L HS	L AMTR/IA	1	MMM
M	M	23/21	d	L Mes	L HS	L AMTR/IA	1	LMM
N	F	41/38	w	L Mes	L HS (like)	L AMTR/IA	1	LLL
O	F	23/5	d	R Mes	R Tumoral lesion	R AMTR/IA	1	MMM
**P**	**M**	**20/11**	**w**	**L Mes**	**Normal**	**L AMTR/III**	**0**	**LLL**
Q	F	30/16	w	R Mes	R HS	R AMTR/IA	1	LLL
R	F	32/15	d	L Mes	Normal	L AMTR/IA	1	LLM
S (*)	**M**	**26/15**	**w**	**Bi L > R**	**Normal**	**R AMTR/III**	**0**	**LLL**
T (*)	F	35/9	w	Bi	R HS (like)	R AMTR/I	1	LLL

M: male; F:female; d: daily; w: weekly; m: monthly; L:left; R:right: Mes: mesial; Lat: Lateral.

T: temporal; Bi: bilateral; HS: hippocampal sclerosis; Bi:bilateral; MS: Mesial sclerosis; A-M: antero-mesial; Like: likely; AMTR: anterior medial temporal resection; Cort: Cortectomy.

(*)Palliative surgery.

The outcome column corresponds to the Engel classification [36]. Gray rows correspond to patients with a surgery outcome other than Engel I.

### Data Preprocessing

All of the analyses carried out in this paper were performed retrospectively. Thus, tailored lobectomy was not based on the results discussed here. An intraoperative ECoG was recorded for 15–20 min using a 32-channel amplifier (Easy EEG II, Cadwell, USA), filtered at 0.5–400 Hz bandwidth with a 50 (±0.5) Hz notch filter and finally downsampled at 200 Hz. Data were exported to EDF and converted to ASCII for further retrospective analysis. Artifact-free epochs lasting from 3 to 5 min were selected by visual inspection. We used consecutive, nonoverlapping windows of 2048 data points (10.24 s at 200-Hz sampling rate) to obtain records of an average number of 50.000 data points for each of the 28 channels. This time window made it possible to include most of the nonstationarities included in the time series. This last fact was assessed by comparing windows averages with whole records analysis. All analysis programs were written in GNU Fortran and R.

### Numerical Methods

**Figure 1:**
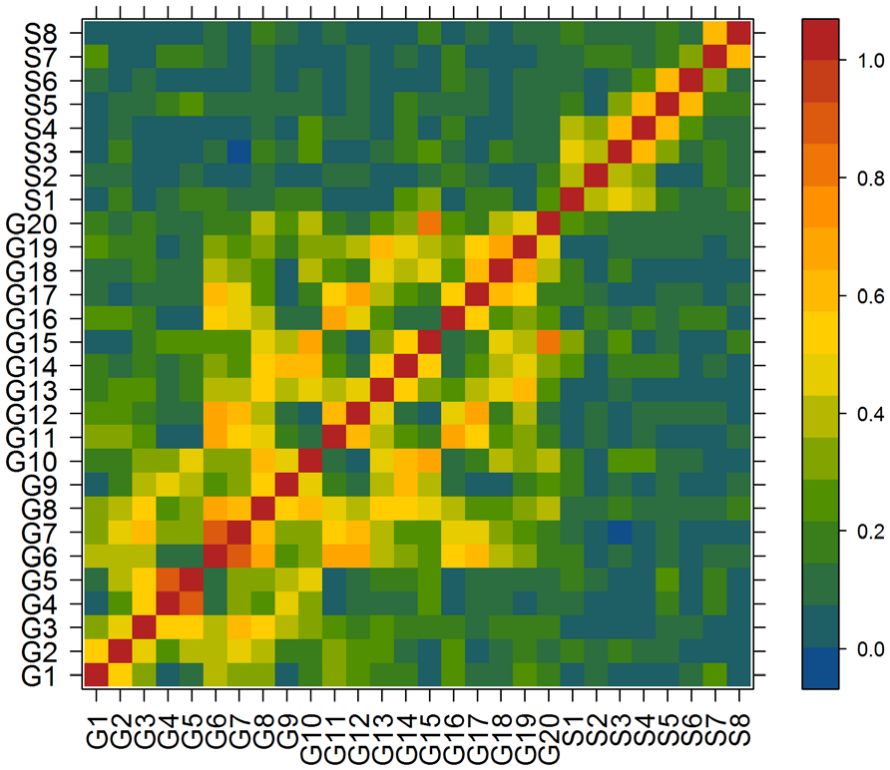
Correlation matrix for the full set of electrodes. Electrodes labeled G1 through G20 belong to the lateral temporal grid; electrodes labeled S1 through S8 belong to the mesial strip. representation corresponds to a single temporal window of patient “G” (Table 1).

In order to estimate interactions between the cortical areas covered by the electrodes, the cross-correlation between each electrode time series was calculated. As shown elsewhere [30], [31], linear cross-correlation performs similar to (and sometimes better than [31]) nonlinear methods in order to estimate functional connectivity, at least when analyzing neurophysiological data. Phase synchronization, through the use of mean phase coherence (see Supporting Information S1) between each pair of electrode’s time series was also calculated in order to compare against the Pearson correlation. The last one will be used as the main functional connectivity estimate throughout the present work. Pairwise interactions between all 28 channels (20 from the lateral grid and 8 from the mesial strip) were calculated in each temporal window of 10.24 seconds. A representative correlation matrix for a specific temporal window is depicted in Figure 1.

As we previously showed [21], LS is an important local centrality measure that seems to be predominantly involved in seizure dynamics as compared with other network characteristics [32], [33], at least from a broad-band point of view.

At the location of each of the 28 electrodes, LS was calculated so that for the cortical area located under the *i-*electrode.
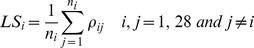
(1)where *ρ_ij_* is the absolute value of the Pearson correlation coefficient between electrode *i* and its nearest neighbor *j.* The first neighbors are the closest *n_i_* electrodes of *i*, including those in diagonals. Therefore, for an electrode in the center of the grid, *n_i_* = 8; in a corner, *n_i_* = 3. In the strip, however, only two cases are possible, *n_i_* = 1 (borders) and *n_i_* = 2 (remainder). Thus, the distribution of LS was obtained across the cortical areas covered by the electrode grid and strip. In the Supporting Information S1 we explain how to better estimate LS, Equation (1), by using the Fisher r-to-Z transformation.

Neurophysiological records from epileptic patients always include paroxysmal activity in the form of spikes or sharp waves. Those paroxysmal patterns occupies at most 13% of the total record in ECoG data ([34] and references therein). As we have shown recently [34], [35], for levels of correlation greater than 0.6/0.7, the influence of spikes over the background synchronization is less than 5%. In the present case the maximal average (across all patients and all channels) value of Pearson correlation coefficient is 0.7312±0.091 and minimal value is 0.2882±0.1278. Because we are mainly interested in the highest values of synchronization, we can assume that paroxysmal activity will not influence considerably the synchronization values and therefore the LS defined in Equation (1).

### Statistical Evaluation

The EZ is a theoretical concept [2], although it is operationally defined, and must be considered as resected if the patient is seizure-free after surgery. Because there is no diagnostic modality which allows the EZ to be determined directly, outcome of surgery is the only “measure” of EZ localization. In fact, outcome of surgery is the parameter against which any other hypothesis should be tested. However, when dealing with the role played by several areas instead of a single one such as the EZ, a statistical approach is necessary.

In order to quantitatively assess the outcome of surgery, we categorized the standard Engel classification [36] on two different levels. One level, labeled as “1”, corresponds to the case of a successful surgical intervention and is thus equivalent to Engel class I, i.e., a patient free from disabling seizures after surgery. The other cases, labeled as “0”, correspond to other surgical outcomes, namely, Engel classes II, III, and IV, i.e., patients not free from disabling seizures after surgery (see Table 1, column entitled “Success”). Consequently, the whole set of surgical outcomes gives a binary sequence (column “Success” in Table 1), namely:
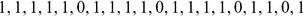
(2)


Instead of a single area, such as the EZ, we analyze several areas, which are localized according to intensity and temporal stability of LS. The simplest way to look at both properties simultaneously is using the coefficient of variation, CV = σ/µ, where σ is the temporal variability and µ the mean value of LS (Equation 1) across the temporal windows. For a single patient X, a set of 28 CVs are obtained, one for each electrode position. Areas with intense LS and low temporal fluctuations are quantified by lower values of CV. A ranking of CVs is constructed, thus:
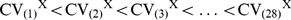
(3)where CV_(1)_^X^ corresponds to the *i*-electrode position of patient X with smallest CV of LS, calculated in the whole record.

This ordering makes it possible to implement the statistical evaluation. Let us assume that we wish to evaluate involvement in seizures of only the first minimum of CV, namely, CV_(1)_. By performing a selection in every patient
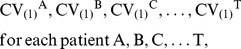
(4)we only need to look at whether locations of these areas were resected or not during surgery. If CV_(1)_ was resected, we assign a “1” (success) or a “0” (failure). This can readily formalized with the indicator function χ



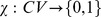
(5)

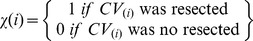
(6)


Therefore, the following binary sequence is obtained.
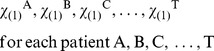
(7)


that can be compared with the surgery success sequence (2) using a correlation. A correlation between two binary sequences can be established using the Pearson coefficient, which in this particular case is sometimes known as the φ coefficient [37].

The same procedure is repeated for the next case considering the first two minima in the sequence (3), namely, CV_(1)_ and CV_(2)_. Three outcomes are possible: i) The case that both areas were resected during surgery, ii) the case that only one area was resected during surgery, and iii) the case that neither was resected during surgery. We assign “1” to case i), “0.5” to case ii), and “0” to case iii). A sequence of 20 ternary numbers {0, 0.5, 1} is thus obtained and can be correlated with the surgery success sequence (2) by defining an indicator function such as (5), but for three elements {0, 0.5, 1}. The same procedure is performed for the case of the three, four and five first minima in (3). For the last case, for instance, the six possible results in every number in the sequences are {0, 0.2, 0.4, 0.6, 0.8, 1}, which correspond to the following resected areas: 0 (none), 0.2 (one), 0.4 (two), 0.6 (three), 0.8 (four), and 1 (all five).

In order to evaluate the goodness of fit of the correlation calculations performed, we used a bootstrapping approach and introduced a surrogate data set for each of the above five cases. Basically, we replaced each sequence like (7) with a surrogate sequence, which was obtained by considering a specific area, such as G1 for instance, instead of minima values of CV. This procedure was implemented for each of the five cases considered.

### Surrogate Data Sets

A bootstrapping approach was used to assess the statistical significance of the correlations obtained. We introduced surrogate [38], [39], [40], [41] data sets for each of the five cases as follows. We replaced the first case, CV_(1)_ with a random area, instead of the first minimum of CV. Specifically, we consider each of the 28 areas corresponding to each electrode as surrogates of CV_(1)_. Thus, we first considered the area of electrode G1 and determined whether this area was resected during surgery in each of the 20 patients, thus generating a surrogate binary sequence of 20 binary numbers. A correlation with (2) is then performed and a surrogate correlation estimate is calculated. The same procedure is then repeated for the remaining electrodes (G2 to S8), thus generating 28 correlation estimates. The distribution of these correlation estimates is used to test the actual correlation estimate obtained between resection of CV_(1)_ and success of surgery.

The same procedure is used for the cases of two, three, four, and five areas. In each case, all possible combinations of positions are calculated. For the case of two areas, we evaluated all possible combinations of two different positions during surgery (G1G2, G1G3, G1G4, …. G18S5, …, S7S8) and established a total of 378 data sets. For each combination we determined whether these areas were resected during surgery, obtaining 0, 0.5, or 1, as in the case of the original data. Thus, 378 data sets, each with a sequence of 20 values (0, 0.5, or 1) will be correlated with the surgery success sequence (2) to generate 378 values of surrogate correlations. The explained procedure is then repeated for the case of three areas (3276 values), four areas (20475 values), and five areas (98280 values). In other words, in each case, n-combinations of a set of 28 elements,
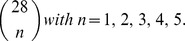
are calculated for the following combinations,



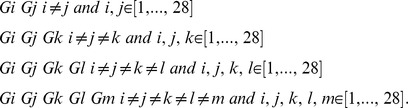
where, for the sake of notational simplicity, we identified G21 to G28 with S1 to S8.

Based on these surrogate data sets and assuming a normal distribution, a statistical evaluation of the original correlation results can be accomplished using Z-scores.

## Results

### Low Variability of High LS Areas

Correlation matrices were obtained for all patients in each temporal window of 2048 data points (10.24 sec.); 30 windows were typically used in each multivariate record. Figure 1 shows a representative correlation matrix in a specific temporal window, corresponding to time series displayed in Figure S1. Two main regions of intense interactions can be observed. The first corresponds to grid electrodes G1–G20 and the second to mesial strip electrodes S1 to S8. The difference between the intra- and inter-region interactions is readily apparent. Intra-lateral interactions (G1–G20 on both axes) and intra-mesial interactions (S1–S2 on both axes) are stronger than the inter-region and lateral-mesial interactions (G1–G20 horizontal axis and S1–S8 vertical axis).

**Figure 2:**
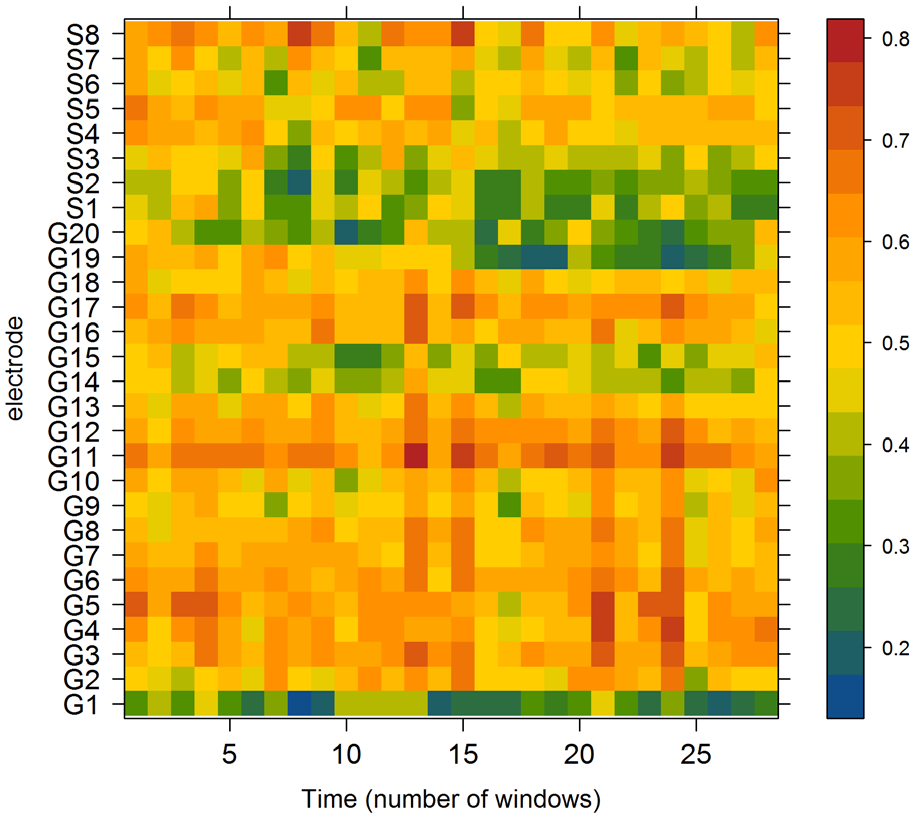
Temporal evolution of LS for each electrode in a specific patient. Each temporal window lasts 10.24 seconds. Representation corresponds to patient “G” (Table 1).

Using the correlation matrix for each temporal window as an estimate of the functional connectivity between each cortical area, LS is calculated based on Equation (1). As we have shown elsewhere [21], the maxima of this measure seem to be involved in seizures. Figure 2 displays the behavior of LS for each cortical area (y-axis) as a function of time (x-axis), where time is expressed in units of temporal windows of 2048 data points.

**Figure 3:**
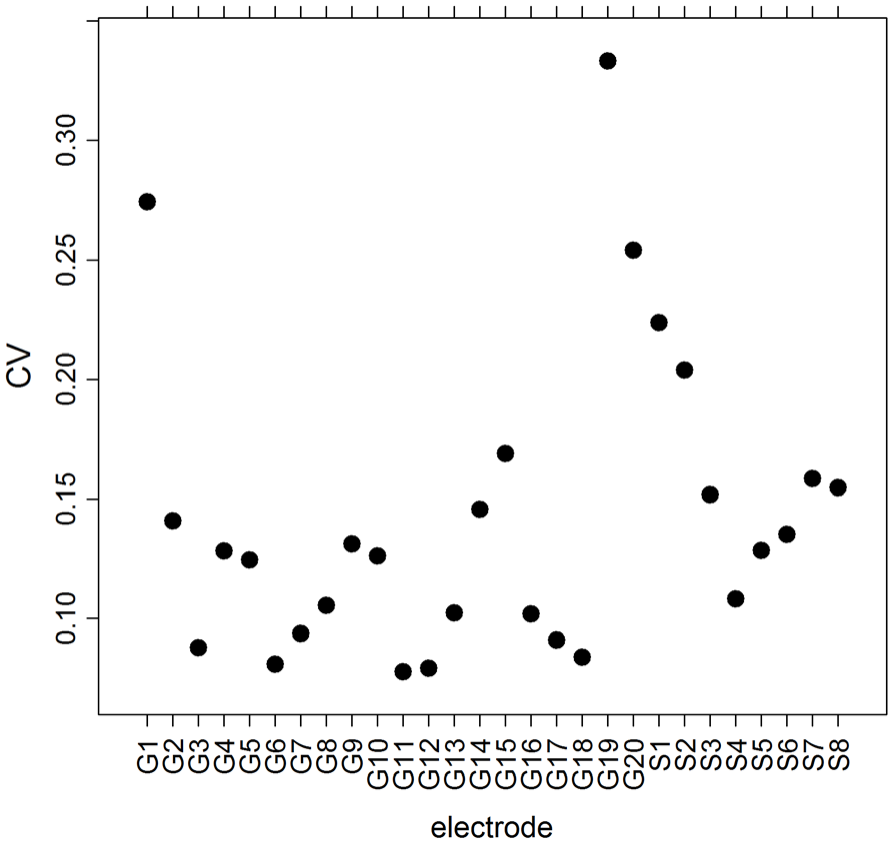
CV for the evolution of LS throughout the recording for each electrode. Data correspond to the activity displayed in Figure 2 corresponding to patient “G”.

Figure 2 shows the existence of a rather diverse distribution of LS values, ranging from very low values (e.g., in electrode G1) to high values (e.g., in electrode G11). Given the definition of this measure, the value of Equation (1) is independent of the specific location in the grid or on the strip, because it is always divided by its connectivity. In the case of G1 for instance, LS is calculated from its first three neighbors, G2–G6–G7, whereas in the case of electrode G11, LS is calculated from its interactions with G16–G7–G12–G16–G17. Close examination of Figure 2 reveals that some areas display greater temporal variability than others in terms of LS dynamics. Areas with higher LS values seem to behave more stably than areas with lower values. This is particularly evident when comparing LS in electrode G11 with, for instance, LS in electrode G15. The first almost always attains values greater than 0.6, whereas electrode G15 shows greater variability, from a value of LS close to 0.3 (window 10) to values close to 0.5. In order to investigate this observation, we calculated temporal mean values (µ) and temporal standard deviations (σ) throughout the recording for each LS location. In Figure 3 we plot the temporal coefficient of variation (CV = σ/µ) for the evolution of each LS area over time. This figure seems to confirm that areas with higher LS values simultaneously display lower variability, quantified by a low σ and, therefore, by a low CV. Moreover, areas with lower CVs, namely G11, G12, and G16, are very close to one another in the grid.

Intense LS clusters are involved in seizure generating circuits, because complete or part resection of these clusters eliminates postoperative seizures. In this particular case, patient “G” in Table 1, these three high LS clusters were resected during the surgery, resulting in an Engel class I, free of postoperative seizures.

These areas are characterized by simultaneously having the highest LS and showing this behavior in a very stable fashion. We call them local stable synchronization areas (LSSA).

### LSSAs Resection and Outcome of Surgery

Calculations of LSSA for every electrode in each patient’s record were performed in the following way. The temporal mean values and standard deviation of LS were estimated across the windows for every electrode, and minima were identified for the CV. Since differences between minima of CV are sometimes subtle (e.g., for G11 and G12 in Figure 3), we chose the first five minima for each patient and compared them with those of the areas resected during surgery.

**Figure 4:**
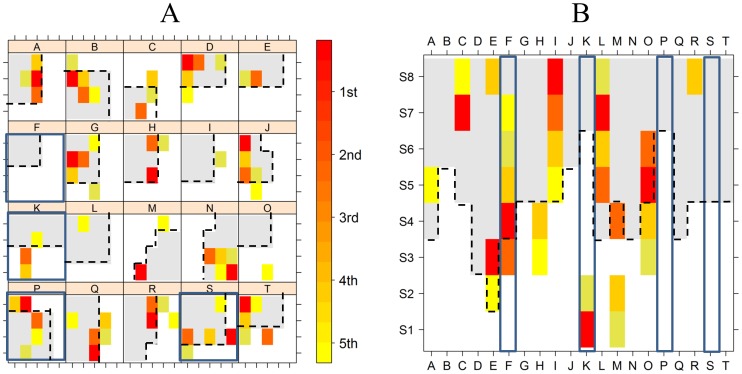
Localization of CV_(1)_ to CV_(5)_ and resected tissue in each patient (Pearson correlation coefficient). (A) Cortical grid electrodes: Gray areas represent the approximate lateral cortical tissue resected during surgery. Superimposed, CV minima are displayed according to the key bar (right): CV_(1)_ = 1st, CV_(2)_ = 2nd, CV_(3)_ = 3rd, CV_(4)_ = 4th, and CV_(5)_ = 5th. Patients with postoperative seizures are highlighted in blue. G1 is always at the bottom left and G20 at the top right position. (B) Mesial strip electrodes: Same schematic diagram as in panel (A).

Figure 4 shows the distribution of LSSA across the electrode grid and strip and displays a representation of the tissue resected during surgery (gray areas limited by dashed lines). In panel (A), a representation of the grid is depicted for every patient. Gray areas represent the resected tissue, and the colored areas (according to the key bar) represent the locations of the minima CV values in the grid. The position of G1 in each grid is always at the bottom left and G20 is always at the top right. For instance, in patient A, the resected area encompasses electrodes G6, G7, G8, G11, G12, G13, G16, G17, and G18, and LSSA are located in CV_(1)_^A^  =  G13, CV_(2)_^A^  =  G8, CV_(3)_^A^  =  G18, CV_(4)_^A^  =  G12, and CV_(5)_^A^  =  S5. Moreover, patients for whom surgery fails (“0”) are highlighted in blue (patients F, K, P, and S). In panel (B), mesial strips are represented in the same fashion as the grids in panel (A). In patients without postoperative seizures, greater parts of the CV minima were resected during surgery (colored areas overlapping with gray areas). On the other hand, in patients with postoperative seizures, several colored areas are visible outside the gray ones.

Identical representation is depicted in Figure S2 when functional connectivity between electrodes is calculated by using the r-to-Z transformation for average correlation. The cases of phase synchronization and r-to-Z phase synchronization are depicted in Figures S3 and Figure S4 respectively. Differences between these cases will be quantified when comparing each one against the corresponding surrogates (see below),

### No Relationships between LSSA and SOZ

It is noteworthy that no association was found between LSSA locations and SOZ. Table 1 shows the SOZs in the v-EEG column, as revealed by traditional ictal signal analysis. V-EEG (see Methods) analysis allows to determine sublobar lateralization of the SOZ, i.e., whether the SOZ is at the lateral or at the mesial side of the epileptogenic temporal lobe. More accuracy can also be achieved by determining in which electrode the seizure begins. However, the information provided by the intraoperatory ECoG mesial strip does not correlate with those given by the FOE because both are placed in different position and therefore, record activity from different mesial structures. Thus it is impossible to compare precisely SOZ location, determined by v-EEG with LSSA locations which are determined by ECoG. However, it is still possible to compare sublobar lateralization in both SOZ and LSSA. In Table 1, the column “CV loc” display sublobar lateralization of the first three CV minima for each patient. This information should be compared with the v-EEG column in the same table. For instance in patient A, sublobar lateralization of SOZ is mesial but sublobar lateralization of the first three CV is lateral in every one. One can easily check that there is no relation between both localizations. In fact, the unique patient with lateral SOZ (patient F) has its first three CVs in the mesial side.

In most cases (17 patients) with mesial SOZ, there exist very different patterns of LSSA locations, without a definite correlation with the SOZ.

### Comparison against Surrogate Data

A statistical analysis was carried out as explained in the Methods section. A correlation was established between success of surgery (2) and resection of CV_(1)_. The other four cases were also considered, from the case of the first two minima CV_(1)_ and CV_(2)_ to the case of the first five minima CV_(1)_, CV_(2)_, CV_(3)_, CV_(4)_ and CV_(5)_.

Table 2 shows the estimated correlation estimate between outcome of surgery (2) and LSSA resection. The maximal correlation estimate is obtained for the case of only one minimum, which corresponds to the LSSA marked with the stronger red color in Figure 4. In this case, CV_(1)_ was resected in every patient except K, P, and S. Sequence (7) therefore takes the following value:
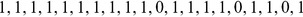
(8)


**Table 2:** Estimates, p-value and Z-scores in the four methods of synchronization between the resection of minima and seizure success.

Number of CV minima	Correlation			Correlation (r-to-Z)			PS			PS (r-to-Z)		
	Est	p-value	Z	Est	p-value	Z	Est	p-value	Z	Est	p-value	Z
CV_(1)_	0,840	3,5E-06	3,87	0,577	0,00769	2,61	0,667	0,00133	3,04	0,140	0,55599	0,52
CV_(1)_, CV_(2)_	0,829	0,00001	4,08	0,590	0,00614	2,85	0,514	0,02029	2,47	0,729	0,00027	3,56
CV_(1)_, CV_(2),_ CV_(3)_	0,663	0,00143	3,32	0,443	0,05020	2,15	0,585	0,00678	2,90	0,583	0,00698	2,90
CV_(1)_, CV_(2),_ CV_(3)_, CV_(4)_	0,464	0,03927	2,31	0,499	0,02509	2,50	0,452	0,04527	2,25	0,515	0,02011	2,59
CV_(1)_, CV_(2),_ CV_(3)_, CV_(4)_, CV_(5)_	0,344	0,13767	1,68	0,414	0,06975	2,08	0,414	0,06975	2,08	0,465	0,03873	2,37

**Figure 5:**
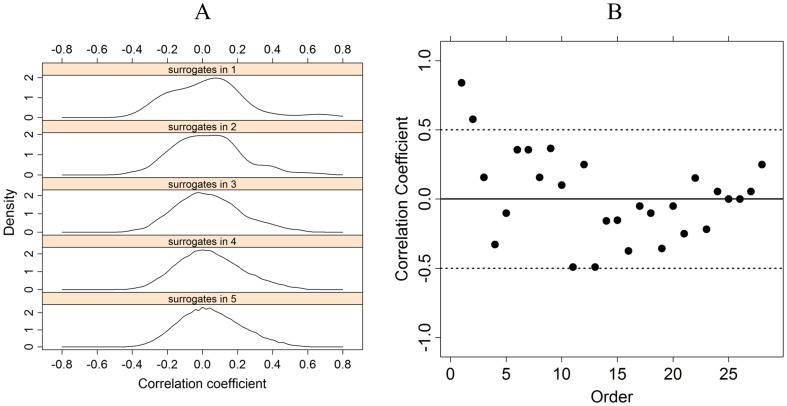
Surrogate distributions and CV_(i)_ correlations. (A) Density distributions of correlation coefficients for surrogate data. The upper panel corresponds to the case of a single random location. The lower panel corresponds to the case of combinations of two, three, four, and five. See text for explanation. (B) Correlation coefficient between seizure success sequence (2) and CV_(i)_ resection, for 1 = 1, …28.

The statistical assessment was performed using surrogate data. As an example, consider the case of a fixed location in the grid, for instance G1. By looking at G1 (the left down corner in every patient) in Figure 4, the following binary sequence is obtained. 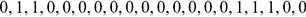
(9)that is, a “0” when G1 was not resected and “1” when it was. The sequence in (9) was then cross-correlated with the surgery success sequence (2) in order to obtain the first surrogate correlation estimate. The same procedure is then repeated for every other position in the grid and strip, with results of the 28 surrogate correlation estimates. The upper panel of Figure 5 A (“surrogates in 1”) displays the approximate distribution of these correlation estimates.

A similar procedure is repeated for two, three, four, and five minima of CV.

Approximate density distributions in each of the five cases are depicted in Figure 5 A. Note the similarity between the distributions and the approximation toward a normal distribution as the number of positions (1 to 5) increases. Consequently, a Z-score can be confidently applied. Results of Z-scores are summarized in Table 2 (Correlation column).

When functional connectivity is estimated by using phase synchronization instead of correlation or by using the r-to-Z transformation in Equation (1) (see Supporting Information S1), CV minima distribution change (compare Figures S2, S3 and S4 with Figure 4). However the changes are mainly in the ordering in the group of the first minima. When applying the same procedure as described above in each one of these cases, namely, r-to-Z correlation, phase synchronization and r-to-Z phase synchronization, the scores are also significant in almost every case. In Table 2 we summarize correlations between surgery outcome and CV minima resection in each one of these cases.

Finally, we calculated the correlation between success of surgery and resection of only one of the CV_(i)_, but for every i = 1, …28, in order to determine whether the first minimum, CV_(1)_, was the only one with particular relevance. As can be seen in Figure 5 B, the maximum correlation value is effectively reached by CV_(1)_ (0.840) followed by CV_(2)_. No other CV_(i)_ seems to be of particular significance.

### LSSA Distributions

**Figure 6:**
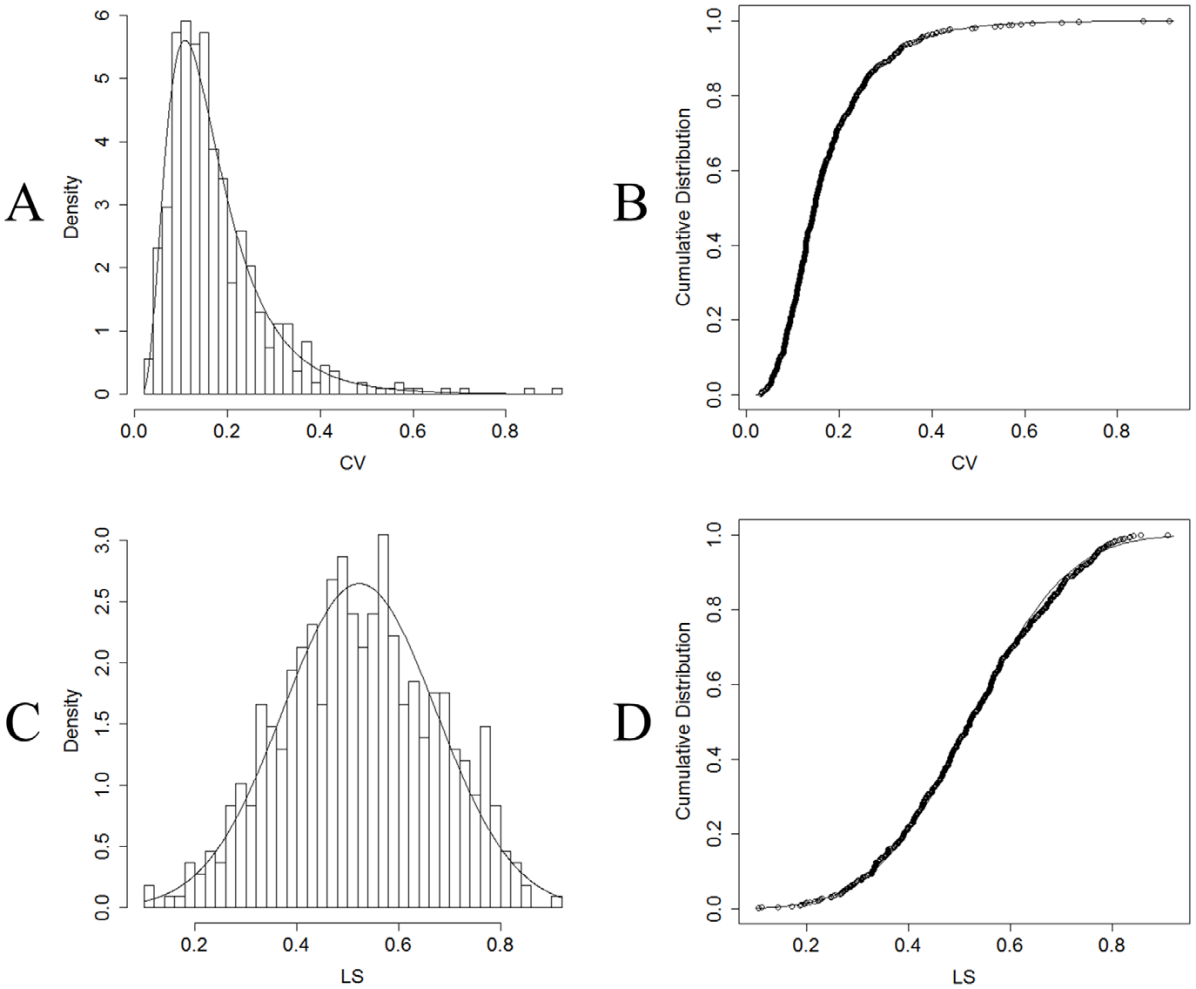
LS and CV distributions. (A) Density and (B) cumulative distributions of CV for every electrode from every patient. A lognormal distribution is superimposed (meanlog  = −1.9081476, sdlog  = 0.5620142). (C) Density and (D) cumulative distributions of mean LS values for all the electrode of each patient. A normal distribution is superimposed (mean  = 0.5228, standard deviation  = 0.1507480).

In order to complete our analysis, we addressed the association between LS areas and LSSA distributions. We used the set of values for the entire sample (approximately 28×20 = 560 values). For the CV distribution, the best fit for a theoretical density distribution corresponded to a lognormal distribution (Kolmogorov-Smirnov goodness-of-fit  = 0.0282763 and Anderson-Darling goodness-of-fit  = 0.391705, both at the 5% significance level), with meanlog  = −1.9081476 (±0.02416) and sdlog  = 0.5620142 (±0.01708). Theoretical and empirical distributions are displayed, respectively, in Figure 6 A and 6 B. In the case of LS values, the best fit for the empirical distribution was a normal distribution (Kolmogorov-Smirnov goodness-of-fit  = 0.03147972 and Anderson-Darling goodness-of-fit  = 0.6492742, both at the 5% significance level), with a mean  = 0.5228 (±0.00648) and standard deviation  = 0.1507480 (±0.004581). The theoretical and empirical distributions are displayed, respectively, in Figure 6 C and 6 D.

**Figure 7:**
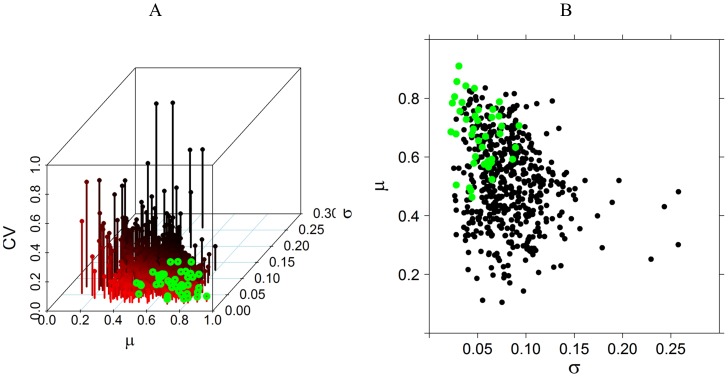
CV, µ and σ relations. (A) Three-dimensional plot showing the relations between mean (µ), deviation (σ) of LS, and CV. The first three lower CVs for each record are coloured in green. (B) Projection of the three-dimensional plot of (A) in the µ-σ plane.

Lastly, the association between LS values and CV values is illustrated in Figure 7. The whole set of values is depicted in Figure 7 A as a function of LS, standard deviation (σ), and CV in a three-dimensional plot. We highlighted (green) the first three CV minima for each patient in the same fashion as in Figure 4 for the first five minima. In order to present the distribution of the first CV minima with respect to the whole population, Figure 7 B shows a projection plotted in the µ-σ plane of these points.

Figure 7 B addresses the question of whether LSSA, which is the quotient of two quantities, arises as a combination of both high LS areas and low variance, because most low CV values are located at the top left of the LS-σ plot.

The definition of CV is the inverse of the signal-to-noise ratio, SNR = µ/σ, a parameter that is widely used in digital signal and imaging processing. According to the Rose criterion, images with SNR>5 can be considered deterministic features. SNR>5 are equivalent to CV<0.2. Figure 6 A shows that areas with such CV belong to the main body of the lognormal distribution. The possible implications of this observation in the seizurability of the whole epileptic network are discussed below.

## Discussion

We demonstrate the existence of intense LS areas in the lateral and mesial cortices of TLE patients. These areas, which simultaneously show the most intense LS and the most stable behavior, are associated with seizures. Statistical analysis showed that when these areas were removed during surgery, patients remained free of postoperative seizures. On the contrary, when these areas were not resected during surgery, the patient continued to suffer from postoperative seizures (Engel class other than I). Testing to determine whether our results could be replicated using randomly chosen areas was negative.

As stated in the introduction, several groups have already reported the existence of cortical areas of high local synchronization. In [7] it is shown in nine patients with partial epilepsies (temporal and extratemporal) the existence of high local, and stable, synchronization areas measured using phase synchronization. Although the small sample presented in that case, they also speculate that resection of these synchronization areas will contribute to seizure control. However, the existence of a relationship between SOZ and local synchronization areas could not be addressed in that case. Similar results and conclusions, although in a greater and more homogenous patient sample (29 TLE patients) were achieved in a previous work of our group [21]. Dauwels et al. [22] have also shown the existence of high local synchronization areas in a sample of 6 patients, although in that case, SOZ seems to be correlated with local synchronization areas.

The results presented here in a homogenous sample of TLE patients, put a step forward to the previous findings. On the one side we have shown that LSSA areas display a remarkable temporal stability with implications we will discuss below. On the other side, we have shown that LSSA do not correlate with SOZ, an issue not fully assessed in the past. By using a novel surrogate methodology, we have also shown that seizure control could be achieved through resection of LSSA.

### Patchy Synchronized Activity in Epileptic Cortical Organization

ECoG electrodes record a spatial summation of dendritic activity produced by ionic currents of pyramidal cells. These recordings characterize activity from local field potentials of a single or a few macrocolumns in the cortex. At this level, therefore, detailed information on the underlying neuronal wiring is lost and trying to identify/classify anatomical connectivity patterns [42] from this mesoscopic information would certainly be misleading. However, areas with intense interictal LS indicate considerable underlying activity, owing to a denser connectivity pattern and/or stronger connection strength. Their existence therefore implies a heterogeneous connectivity distribution which has also been described in immunohistochemical, genetic, and electrophysiological studies [43], [44], [45], [46]. As shown in numerical simulations [47], the presence of high LS areas, or hubs, in hippocampal granule cells favors hyperexcitability of the whole network. Similar results have also been shown in pyramidal cells in mammals [48], [49]. Synchronization hubs have been recently described [50] in heterogeneous networks as nodes, highly connected, and displaying local synchronization while the rest of the network remains unsynchronized. These synchronization hubs may lead to a hierarchical transition toward a global synchronization with hubs synchronizing first [50], [51]. One can interpret the results of the present work in the light of the aforementioned theoretical findings. The existence of a few areas, whether mesial or lateral, with high local connectivity seems to favor seizurability in the epileptic network. This hypothesis is also in line with the clever work of Schevon et al. [7] in which they advance similar conclusions.

### Nonrandom Characteristics of LSSAs

The stable character of these synchronization areas plays a central role in seizurability. As shown recently [19], in networks with time-fluctuating links, small-world behavior would appear as a consequence of the presence of time-persistent nodes in the underlying network. In our case, intense LS areas simultaneously display the lowest temporal variability. A considerable difference exists between determination of (mean values of) intense LS areas and LS areas with a low CV, because both stability and intensity are encompassed in the latter. Lower values of CV (<0.1), such as those displayed by LSSA areas, strongly indicate a nonrandom feature in its dynamics, as does the lognormal distribution of CV when compared with the Gaussian distribution of LS. A hypothesis that merits further attention is whether the CV distribution displayed on the epileptic side, as in Figure 6 A, remains approximately invariant on the nonepileptic side or in healthy patients. If the strong and stable character of the LSSAs plays a central role in seizure appearance, a shift in the CV distribution toward higher values of CV would render the network less prone to seizures.

### LSSAs Patterns Facilitate Seizure Propagation

The kind of mechanisms under which these synchronization hubs play a central role in epileptogenesis can be determined by studying how a seizure develops. A seizure begins to spread from the onset area to distal cortical areas as synchronization increases [52]. During development of the seizure, decorrelation dominates the first half, with an increase in correlation during the second half. Two hypotheses have been advanced to explain the dynamic aspects of seizure development. One states that desynchronization is a preexistent state of cortical activity, which, perhaps through a transient synchrony mechanism, could favor development of the seizure. The other postulates that desynchronization during development of the seizure is mainly due to delays in neural communication [53]. The results presented here clearly favor the first hypothesis. Epileptic networks contain several areas with very different interictal synchronization states which favor the development of seizures at different rates. The fact that propagation of the seizure to distant areas is generally much faster than the predicted horizontal intracortical rate of 6–18 cm/s strongly suggests the existence of several routes, instead of a single intracortical spread [53]. Propagation through white matter and subcortical nuclei–mediated spread may enable the seizure to reach distant cortical areas very fast. Ictal information reaching areas of high local synchronization may enhance the synchronizability of the whole network, giving rise to the onset of a seizure.

## Supporting Information

Figure S1 **Representative record of 10 seconds of ECoG time series:** (**A**) **Grid electrodes.** (**B**) **Strip electrodes.**
(TIF)

Figure S2 **Localization of CV_(1)_ to CV_(5)_ and resected tissue in each patient (r-to-Z transformation over the Pearson correlation coefficient).** (A) Cortical grid electrodes: Gray areas represent the approximate lateral cortical tissue resected during surgery. Superimposed, CV minima are displayed according to the key bar (right): CV_(1)_ = 1st, CV_(2)_ = 2nd, CV_(3)_ = 3rd, CV_(4)_ = 4th, and CV_(5)_ = 5th. Patients with postoperative seizures are highlighted in blue. G1 is always at the bottom left and G20 at the top right position. (B) Mesial strip electrodes: Same schematic diagram as in panel (A).
(TIF)

Figure S3 **Localization of CV_(1)_ to CV_(5)_ and resected tissue in each patient (mean phase coherence of phase synchronization).** (A) Cortical grid electrodes: Gray areas represent the approximate lateral cortical tissue resected during surgery. Superimposed, CV minima are displayed according to the key bar (right): CV_(1)_ = 1st, CV_(2)_ = 2nd, CV_(3)_ = 3rd, CV_(4)_ = 4th, and CV_(5)_ = 5th. Patients with postoperative seizures are highlighted in blue. G1 is always at the bottom left and G20 at the top right position. (B) Mesial strip electrodes: Same schematic diagram as in panel (A).
(TIF)

Figure S4 **Localization of CV_(1)_ to CV_(5)_ and resected tissue in each patient (r-to-Z transformation over the mean phase coherence of phase synchronization).** (A) Cortical grid electrodes: Gray areas represent the approximate lateral cortical tissue resected during surgery. Superimposed, CV minima are displayed according to the key bar (right): CV_(1)_ = 1st, CV_(2)_ = 2nd, CV_(3)_ = 3rd, CV_(4)_ = 4th, and CV_(5)_ = 5th. Patients with postoperative seizures are highlighted in blue. G1 is always at the bottom left and G20 at the top right position. (B) Mesial strip electrodes: Same schematic diagram as in panel (A).
(TIF)

Supporting Information S1 **Contains: Information related with the coefficient of variation of the Local Synchronization and phase synchronization measure.**
(DOCX)
