# Supplementary figures and images for: Stability of Synchronization Clusters and Seizurability in Temporal Lobe Epilepsy
Source: PLoS One. 2012 Jul 23;7(7):e41799. doi: 10.1371/journal.pone.0041799 (PMC3402406; doi:10.1371/journal.pone.0041799)

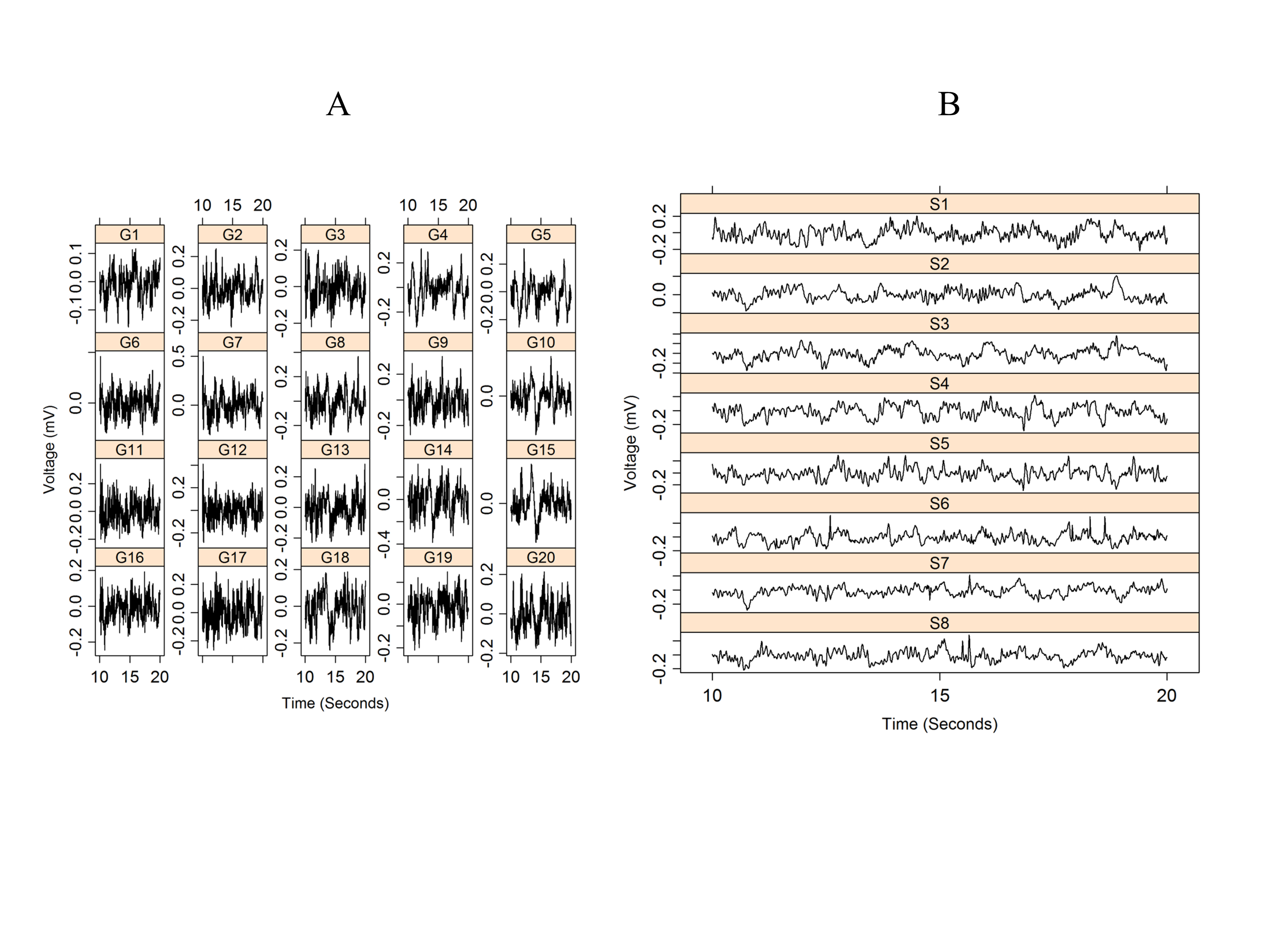

Supplement: Figure S1 — Representative record of 10 seconds of ECoG time series: (A) Grid electrodes. (B) Strip electrodes. (TIF) [file pone.0041799.s001.tif]

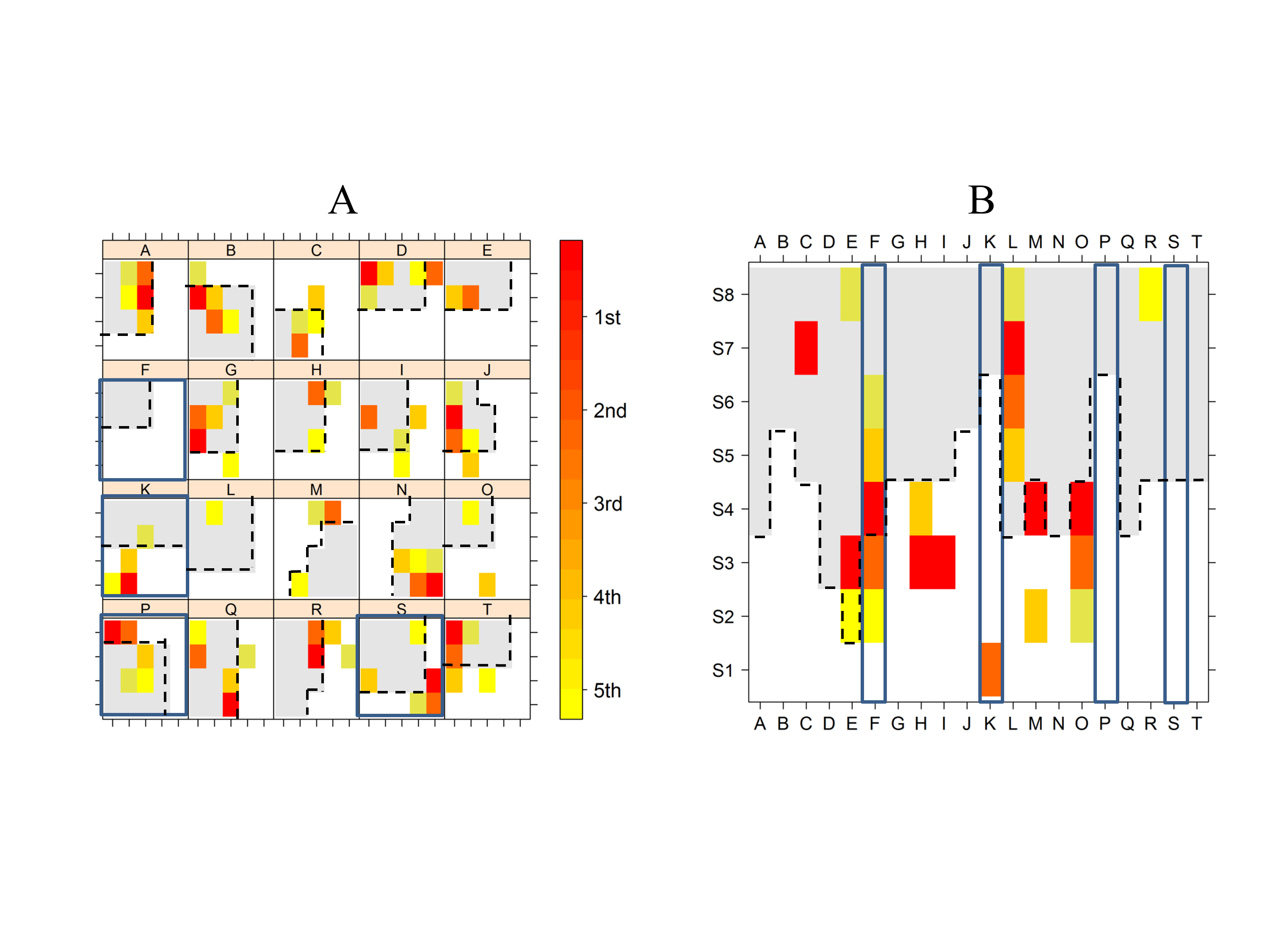

Supplement: Figure S2 — Localization of CV(1) to CV(5) and resected tissue in each patient (r-to-Z transformation over the Pearson correlation coefficient). (A) Cortical grid electrodes: Gray areas represent the approximate lateral cortical tissue resected during surgery. Superimposed, CV minima are displayed according to the key bar (right): CV(1) = 1st, CV(2) = 2nd, CV(3) = 3rd, CV(4) = 4th, and CV(5) = 5th. Patients with postoperative seizures are highlighted in blue. G1 is always at the bottom left and G20 at the top right position. (B) Mesial strip electrodes: Same schematic diagram as in panel (A). (TIF) [file pone.0041799.s002.tif]

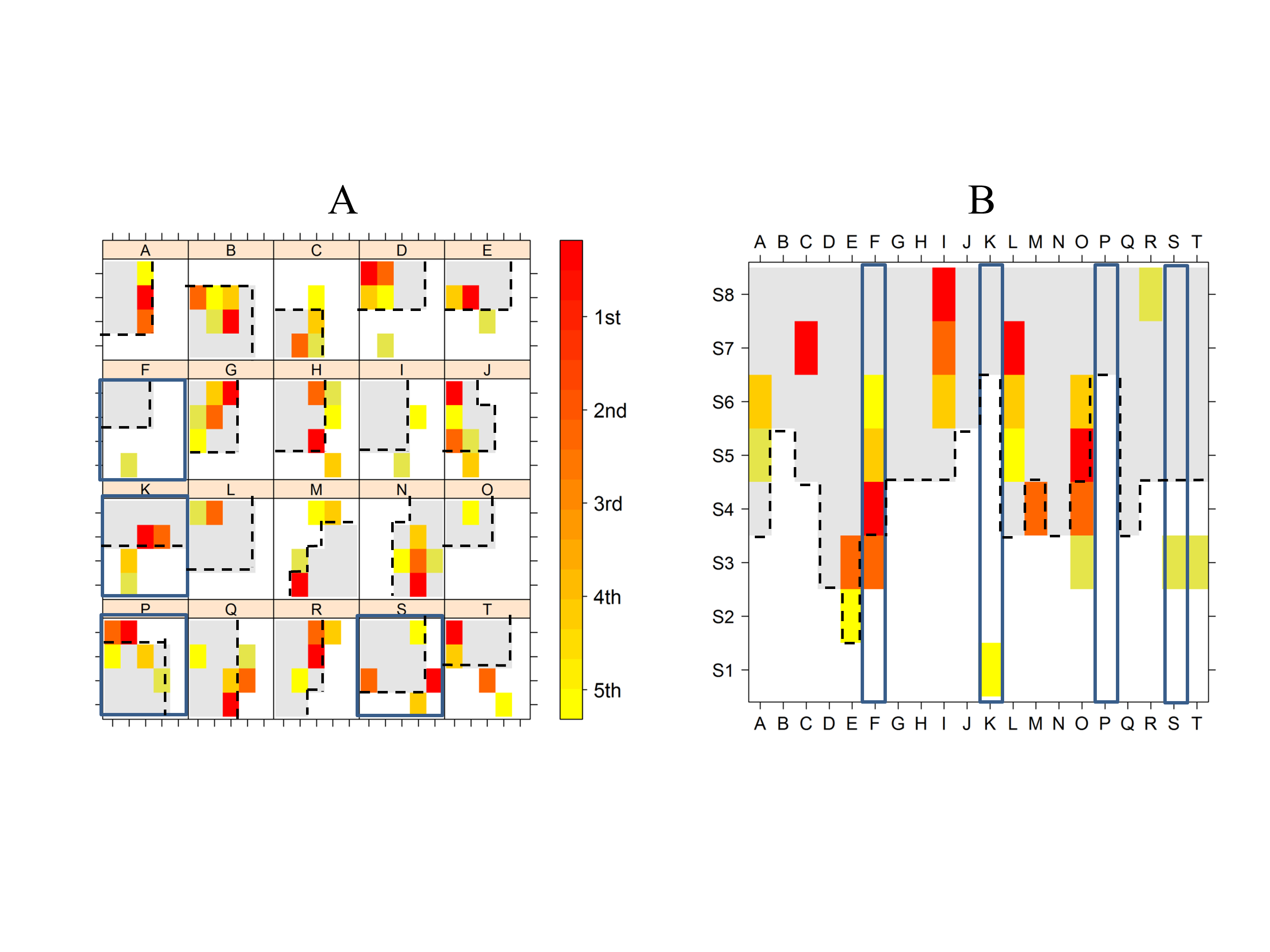

Supplement: Figure S3 — Localization of CV(1) to CV(5) and resected tissue in each patient (mean phase coherence of phase synchronization). (A) Cortical grid electrodes: Gray areas represent the approximate lateral cortical tissue resected during surgery. Superimposed, CV minima are displayed according to the key bar (right): CV(1) = 1st, CV(2) = 2nd, CV(3) = 3rd, CV(4) = 4th, and CV(5) = 5th. Patients with postoperative seizures are highlighted in blue. G1 is always at the bottom left and G20 at the top right position. (B) Mesial strip electrodes: Same schematic diagram as in panel (A). (TIF) [file pone.0041799.s003.tif]

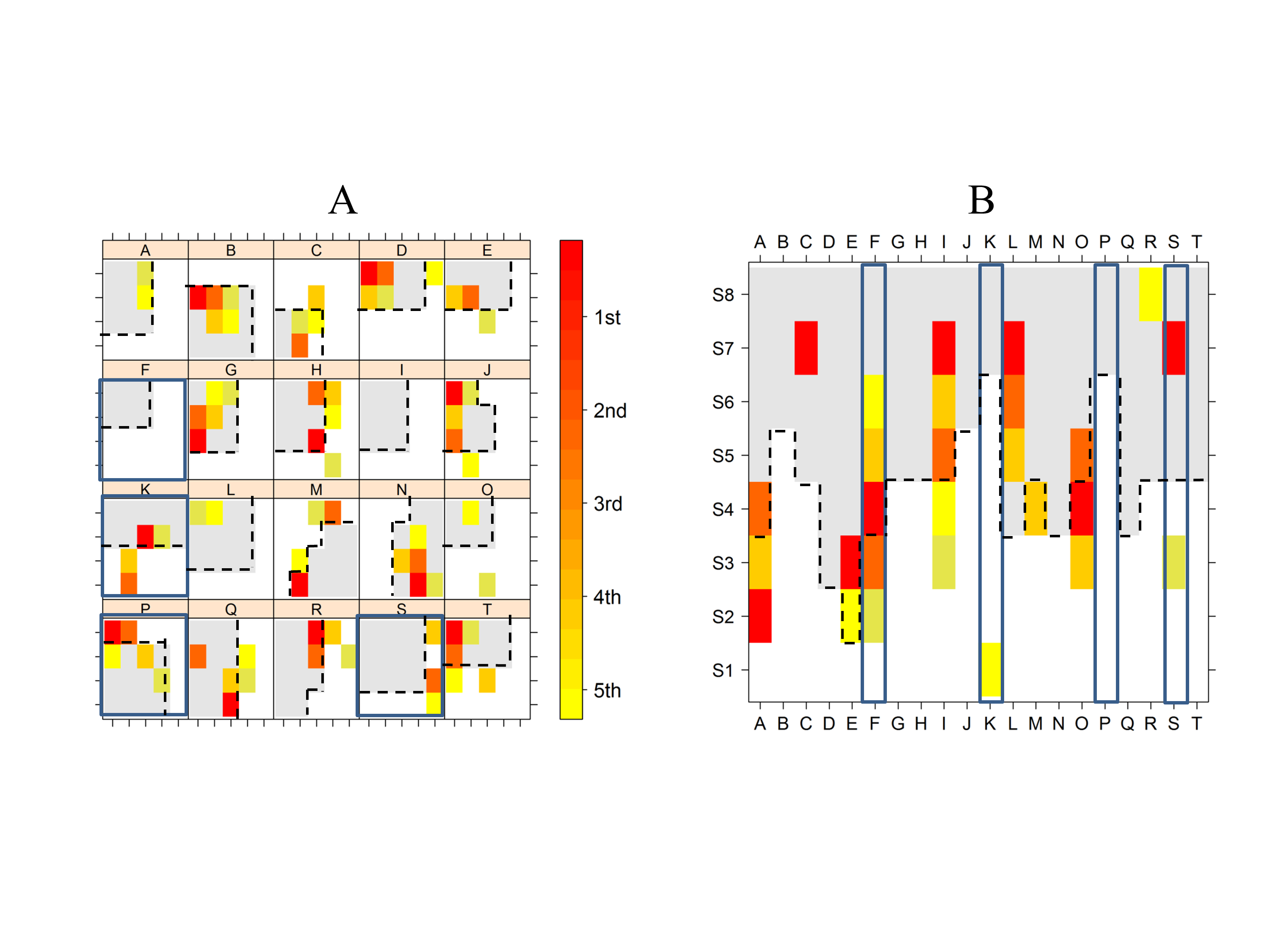

Supplement: Figure S4 — Localization of CV(1) to CV(5) and resected tissue in each patient (r-to-Z transformation over the mean phase coherence of phase synchronization). (A) Cortical grid electrodes: Gray areas represent the approximate lateral cortical tissue resected during surgery. Superimposed, CV minima are displayed according to the key bar (right): CV(1) = 1st, CV(2) = 2nd, CV(3) = 3rd, CV(4) = 4th, and CV(5) = 5th. Patients with postoperative seizures are highlighted in blue. G1 is always at the bottom left and G20 at the top right position. (B) Mesial strip electrodes: Same schematic diagram as in panel (A). (TIF) [file pone.0041799.s004.tif]
