# Supplementary material for: Stability of Synchronization Clusters and Seizurability in Temporal Lobe Epilepsy
Source: PLoS One. 2012 Jul 23;7(7):e41799. doi: 10.1371/journal.pone.0041799 (PMC3402406; doi:10.1371/journal.pone.0041799)
Supplement: Supporting Information S1 — Contains: Information related with the coefficient of variation of the Local Synchronization and phase synchronization measure. (DOCX) [file pone.0041799.s005.docx]

**Supporting Information S1**

*Coefficient of variation of LS*

The idea behind the Local Synchronization (LS), as defined in Equation (1), is to highlights the *local synchronized activity* at the cortical level by summing up correlations between neighbors electrodes,

$LS_{i} = \frac{1}{n_{i}}\sum_{j=1}^{n_{i}} \rho_{ij} \text{ where }\rho_{\text{ij}}=abs(\delta_{ij})$ (S1)

where *δ_ij_* is the Pearson correlation coefficient. Normalizing the sum of correlation by the connectivity (*n_i_*) of each electrode *i* allows to compare LS at different cortical locations. When averaging across several temporal windows, the mean value of LS, considered as a sum of random variables, *ρ_ij_*, is

$\mu_{LS_{i}} = \langle LS_{i}\rangle= \frac{1}{n_{i}}\sum_{j=1}^{n_{i}} \left\langle\rho_{ij}\right\rangle$ (S2)

In order to calculate the coefficient of variation (CV) of *LS_i_*,

$CV_{LS_{i}}=\frac{\sigma_{LS_{i}}}{\mu_{LS_{i}}}$ (S3)

the standard deviation (σ) should be calculated, which again, using Equation (S1) as a sum of random variables *ρ_ij_*, and using the definition of variance, one can obtain that

$\sigma_{LS_{i}}^{2} = \langle LS_{i}^{2}\rangle- \langle LS_{i}\rangle^{2} = \frac{1}{n_{i}}\sum_{j=1}^{n_{i}} \sigma_{\rho_{ij}}^{2} + \frac{1}{n_{i}^{2}}\sum_{j=1}^{n_{i}} \sum_{\begin{matrix} k=1 \\ k\neq j \end{matrix}}^{n_{i}} \mathrm{cov}(\rho_{ij},\rho_{ik})$ (S4)

The second term depends both in the connectivity and in the covariance of the correlation coefficients between neighbor’s sites. However, the second term in Equation (S4) is numerically negligible and CV calculations with or without this term are practically identical.

A second issue which should be considered when using Equation (S1) is the non-additive character of the correlation coefficient. In order to cope with averages of correlation coefficients Fisher [1] has developed a transformation, today known as *r-to-Z transformation* which allows summing up several correlation coefficients. In this way, the first step is to transform the correlation coefficient *ρ_ij_* to a new variable *Z_ij_* such that,

$Z_{ij}=\frac{1}{2}\ln\left[\left.\frac{1+\rho_{\text{ij}}}{1-\rho_{\text{ij}}}\right]\right.$ (S5)

Then, one can average *Z_ij_* values, as in Equation (S1)

$\overline{Z}_{i}=\frac{1}{n_{i}}\sum_{j=1}^{n_{i}} Z_{ij}$ (S6)

Finally, back-converting

$\overline{\mu_{LS_{i}}}=\frac{e^{\overline{Z}i}-e^{-\overline{Z}_{i}}}{e^{\overline{Z}i}+e^{-\overline{Z}_{i}}}$ (S7)

In this way, one can eliminate the underestimation of the true value of the average correlation, caused by the skewness in their sampling distribution.

In our calculations, both the “simple” (Equation (S1)) and the corrected through the r-to-Z transformation (Equation S5-S6-S7)) forms of the LS mean value estimation were used to calculate CV. Same procedure was also applied to the mean phase coherence (see below). Results based in r-to-Z transformation of the Pearson correlation coefficient are displayed in Figure S2. Figure S4 display the equivalent results corresponding the case of r-to-Z transformation of phase synchronization.

*Phase synchronization*

The concept of phase synchronization, introduced by Rosenblum *et al.* [2] in relation to chaotic oscillators has been increasingly used in the last years, especially in the field of neuroscience. It has been also extended to the case of noisy oscillators. The power of the method resides in that it measures the phase relationship, independently on the signal amplitude. In order to evaluate differences between phases in two signals, one must firstly define the *instantaneous phase* of the signal, by means of the analytical signal concept. For a continuous signal $x_{i}(t)$ the associated analytical or complex signal is defined as:

$$z_{i}(t)=x_{i}(t)+i\tilde{x}_{i}(t)=A_{i}(t)e^{i\varphi_{i}(t)}$$

where $\tilde{x}_{i}(t)$is the Hilbert transform of $x_{i}(t)$

$\tilde{x}_{i}(t)=\frac{1}{\pi}p.v.\int_{-\infty}^{\infty} \frac{x(t')}{t-t'}dt'$ (S8)

where *p.v.* stands for (Cauchy) Principal Value. The instantaneous phase is thus,

(S9)

And the phase difference between the two signals can be calculated as (S10)

In order to implement numerically the above definition over two time series $x_{i}(k)$ and$x_{j}(k)$, the mean phase coherence ($R_{ij}$) was introduced [3]:

$R_{ij}=\left|\frac{1}{N_{win}}\sum_{k=1}^{N_{win}} e^{i\Delta\alpha_{ij}(k)}\right|$ (S11)

calculated in the time window *N_win_*, where $\Delta\alpha_{ij}(k)=\varphi_{i}(k)-\varphi_{j}(k)$ is the instantaneous phase difference at the discretized time k. It is clear from Equation (S11) that $0\leq R_{ij}\leq1.$The literature [4] gives useful hints for the numerical calculation of the Hilbert Transform of a time series, i.e. Equation (S8). Calculations done with $R_{ij}$between the two channels *i* and *j* will be called generically *PS.*

*References*

1. Fisher RA (1915) Frequency distribution of the values of the correlation coefficient in samples of an indefinitely large population. Biometrika, 10: 507-521.
2. Rosenblum MG, Pikovsky AS and Kurths J (1996) Phase Synchronization of Chaotic Oscillators, Physical Review Letters 76:1804-1807.
3. Mormann F, Lehnertz K, David, P and Elger CE (2000) Mean phase coherence as a measure for phase synchronization and its application to the EEG of epilepsy patients. Physica D 144:358-369.
4. Rosenblum MG, Pikovsky AS, Kurths J. Schaefer C, Tass, P (2001) Phase synchronization: from theory to data analysis. In F. Moss and S. Gielen, editors, Handbook of biological physics, Elsevier Science, Amsterdam. 297 p.
